# Review of imaging test phantoms

**DOI:** 10.1117/1.JBO.28.8.080903

**Published:** 2023-08-22

**Authors:** Liam B. Christie, Wenhan Zheng, William Johnson, Eric K. Marecki, James Heidrich, Jun Xia, Kwang W. Oh

**Affiliations:** a State University of New York at Buffalo, Sensors and MicroActuators Learning Lab, Electrical Engineering, Buffalo, New York, United States; b State University of New York at Buffalo, Optical and Ultrasonic Imaging Laboratory, Biomedical Engineering, Buffalo, New York, United States

**Keywords:** test phantom, photoacoustic

## Abstract

**Significance:**

Photoacoustic tomography has emerged as a prominent medical imaging technique that leverages its hybrid nature to provide deep penetration, high resolution, and exceptional optical contrast with notable applications in early cancer detection, functional brain imaging, drug delivery monitoring, and guiding interventional procedures. Test phantoms are pivotal in accelerating technology development and commercialization, specifically in photoacoustic (PA) imaging, and can be optimized to achieve significant advancements in PA imaging capabilities.

**Aim:**

The analysis of material properties, structural characteristics, and manufacturing methodologies of test phantoms from existing imaging technologies provides valuable insights into their applicability to PA imaging. This investigation enables a deeper understanding of how phantoms can be effectively employed in the context of PA imaging.

**Approach:**

Three primary categories of test phantoms (simple, intermediate, and advanced) have been developed to differentiate complexity and manufacturing requirements. In addition, four sub-categories (tube/channel, block, test target, and naturally occurring phantoms) have been identified to encompass the structural variations within these categories, resulting in a comprehensive classification system for test phantoms.

**Results:**

Based on a thorough examination of literature and studies on phantoms in various imaging modalities, proposals have been put forth for the development of multiple PA-capable phantoms, encompassing considerations related to the material composition, structural design, and specific applications within each sub-category.

**Conclusions:**

The advancement of novel and sophisticated test phantoms within each sub-category is poised to foster substantial progress in both the commercialization and development of PA imaging. Moreover, the continued refinement of test phantoms will enable the exploration of new applications and use cases for PA imaging.

## 1 Introduction

At present, there is a lack of commercially available test phantoms specifically designed for the photoacoustic (PA) field that encompass all desired parameters and applications of this technology. This review examines the existing phantoms in well-established imaging domains, such as magnetic resonance imaging (MRI), ultrasound, optics, and positron imaging technology. By investigating phantom development and manufacturing in these established imaging fields, valuable patterns can be identified and analyzed. The systematic grouping and organization of the identified patterns, materials, and methods serve as a foundational framework for the development of various phantoms tailored to different PA imaging applications. This approach effectively minimizes the requirement for extensive development of individualized PA phantoms, thereby accelerating the commercialization and research efforts in the field of PA technology.

A diverse array of phantoms exists across various medical imaging modalities beyond PA and herein will be classified as simple (Sec. 2), intermediate (Sec. 3), and advanced (Sec. 4). It is important to note that even advanced phantoms do not possess the capacity to fully replicate the complexity and functionality of the human body.^1^^–^^5^ For example, Ionita et al. provided an overview of the utilization of a three-dimensional (3D) printed vascular phantom for medical device testing and accurate evaluation of a patient’s condition by physicians prior to surgical interventions. The authors acknowledge that although these phantoms can effectively replicate the shape and tactile characteristics of normal human vasculature, there are significant limitations associated with the manufacturing process, materials used, and printer resolution, which hinder the long-term structural integrity of the phantoms across multiple experiments. In addition, it should be noted that these vascular phantoms lack the presence of surrounding tissue, a crucial element that influences the behavior of phantoms during x-ray interventions. Advancing the development of phantoms in the field of PA imaging holds great potential for enabling multi-wavelength imaging of oxygen saturation, capturing dynamic changes in spectral wavelength, improving resolution, and facilitating spectroscopy imaging studies.^6^^,^^7^

To create a phantom that effectively mimics the human body for PA imaging, several important factors must be considered. These factors are summarized in Table 1. In terms of the optical component of PA, it is essential to match the phantom’s characteristics, such as optical absorption, scattering, and refractive index, to those of the target human tissue. This ensures that the phantom exhibits proper behavior when subjected to laser-induced excitation.^8^ Similarly, the acoustic characteristics of the phantom, including speed of sound, impedance, and attenuation, need to be carefully tuned. This enables the phantom to provide an accurate response to the acoustic receiver, replicating the acoustic behavior observed in human tissues.^9^^,^^10^ The mechanical properties of the material, including stiffness, modulus, and density, play a crucial role in ensuring that the phantom exhibits the desired tactile feel, particularly in the experiments requiring physical interaction. For instance, in the case of pulsing arterial phantoms, the material’s behavior under pressurized conditions is of particular importance.^10^ The shape of the phantom is another critical parameter that significantly impacts its behavior. The phantom’s shape can encompass various tissue layers, bone structures, and arterial geometries, among other factors, to accurately represent specific anatomical features. Lastly, the stability and repeatability of the phantom are vital considerations. Phantoms should demonstrate consistent behavior across multiple experiments and yield repeatable results for different research groups. This ensures the reliability and comparability of findings.^11^

**Table 1:** A bulleted list of the major parameters that must be considered to create a phantom that accurately represents the human body under PA imaging.

Types	Major parameters
Optical	• Absorption coefficient
	• Scattering coefficient
	• Refractive index
Acoustic	• Speed of sound
	• Impedance
	• Attenuation
Mechanical	• Stiffness
	• Modulus
	• Density
Tissue structure	• Shape of target tissue area
Stability/repeatability	• Consistent properties over time
	• Repeatable manufacturing of above properties over multiple batches

Not all PA applications necessitate the utilization of test phantoms. The existing phantom technology exhibits limitations in accurately replicating all the intricate effects that drugs exert on the human body. For specific applications, such as PA drug delivery and the assessment of drug responses within the body, *in vivo* testing on living tissues or subjects becomes indispensable.^12^^,^^13^ A study conducted by Khadria et al. pertains to the investigation of the dynamics of ultra-rapid insulin upon injection.^14^ This particular study entailed *in vivo* experimentation utilizing live mice and diabetic swine as subjects, thereby facilitating an accurate comprehension of the actual impact of insulin at the injection site. The effects aforementioned can solely be examined in a living subject due to the incapability of *in vitro* setups to mimic crucial bodily functions, such as blood sugar regulation, inflammation, and insulin absorption.

**Fig. 1:**
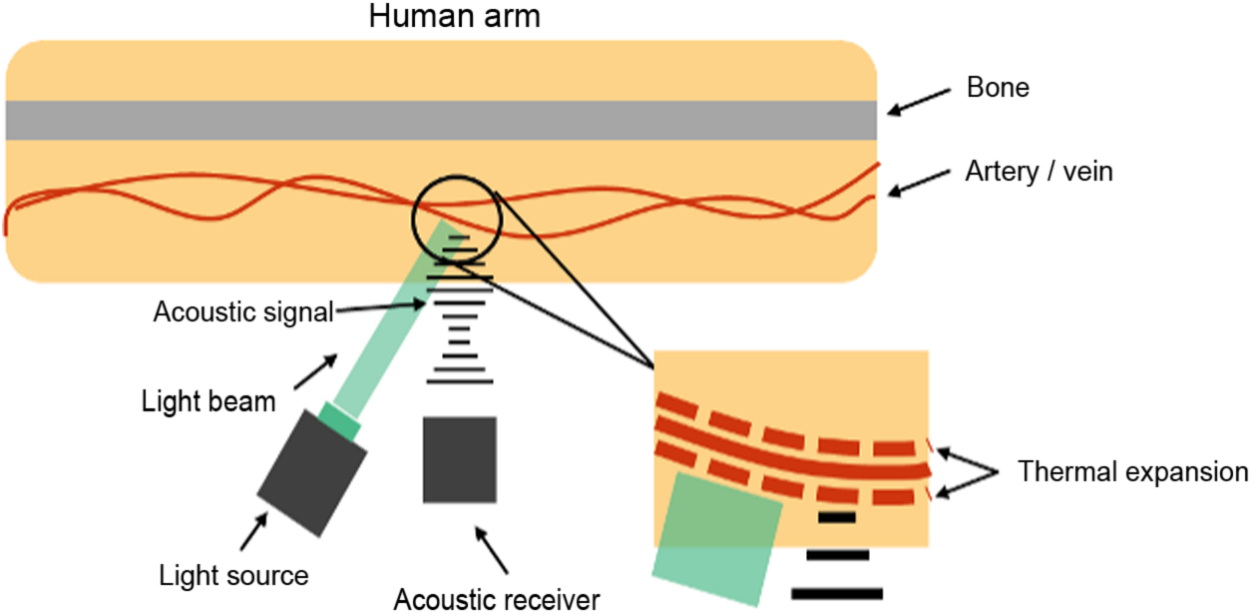
A diagram depicting the basics of PA. A light source shines a focused and pulsing laser into the body. The area of interest goes through rapid thermal expansion creating an acoustic signal. The acoustic signal can then be received by an acoustic receiver.

Photoacoustic tomography (PAT), also known as optoacoustic tomography or thermoacoustic tomography, has experienced significant growth in the past decade and has become a crucial tool in various research areas.^15^ PAT systems have been developed at different spatial scales from nanoscopy to microscopy and tomography.^16^^–^^18^ Different preclinical and clinical imaging applications have also been found for PAT systems. They include cancer detection, functional brain imaging, drug delivery monitoring, and interventional procedure guidance.^19^^,^^20^ In PAT, photons traveling through tissue are absorbed by molecules, leading to the conversion of their energy into heat. This thermal energy generates an initial increase in pressure, which propagates as an acoustic wave. An ultrasonic transducer or transducer array detects the acoustic wave to form an image, which effectively represents the original optical energy deposition in the tissue.^21^ The process is represented in Fig. 1.^22^

Leveraging the considerable optical absorption contrast and low ultrasonic scattering within the tissue, PAT has rapidly emerged as a rapidly advancing biomedical imaging technique.^23^ Compared with ultrasound imaging, PAT provides optical absorption information of the tissue, which functionally complements the structural information from ultrasound. Compared with optical tomographic imaging, PAT achieves a better spatial resolution as it detects weakly scattered acoustic signals.^19^ Furthermore, it is worth mentioning that all the essential features of PAT exhibit a high degree of scalability.^24^ Therefore, PAT offers versatile implementation options, enabling the observation of a given biological process at multiple scales while maintaining a consistent optical contrast. The scalability of PAT is facilitated by its flexible imaging parameters, such as the choice of excitation wavelengths, laser pulse duration, and ultrasound detection methods. The choice of excitation wavelength can be tailored to match the absorption spectrum of the target tissue. However, the imaging depth also plays a role in the wavelength selection, as shorter wavelengths (UV-green) cannot penetrate deep into tissue.^25^ The duration of laser pulses can be adjusted to generate the desired PA signal by considering the stress and thermal confinement of the tissue.^26^ The application of single-element transducers was primarily focused on analog reconstruction, particularly in the field of PA microscopy. On the other hand, transducer arrays found their main application in digital reconstruction, specifically in PA computed tomography.^27^ These parameters can be adjusted to optimize imaging performance based on the specific application and target size.^28^ In addition, advancements in imaging systems, such as the use of sophisticated array designs, high-speed lasers, and deep-learning-based algorithms, contribute to the performance of PAT by enabling faster data acquisition and improved image reconstruction.^29^

## 2 Simple Phantoms

A simple phantom, characterized by minimal manufacturing complexity, typically comprises one or two major components. These phantoms find extensive application across various domains and can be further classified into three sub-categories: simple tube/channel phantoms, naturally occurring phantoms (simple), and simple block phantoms. More comprehensive information regarding the three sub-categories, including the materials employed and their respective applications, is given in Table 2.

**Table 2:** Phantoms identified in the simple category.

Category	Applications	Materials used
Simple tube/channel phantoms	PA resolution measurement at multiple wavelengths	• Carbon fiber strands^30^
		• Pencil lead^31^
	Ultrasonic modalities	• Carbon fiber strands^30^
		• Latex tube
		• Closed-off rubber tube
	MRI	• Delrin plastic^32^
	Pressure transducer measurement	• Silicone tube^33^
	PA imaging	• Rubber tube^34^
		• India ink
		• Latex tube
		• Closed-off rubber tube
		• Methylene blue^35^
Naturally occurring phantoms (simple)	PA resolution quantification	• Human hair
		• Horsehair
		• Leaf^36^^–^^38^
	PA imaging	• Mouse artery^36^
		• Mouse knee
	Ultrasound imaging	• Mouse artery^36^
		• Mouse knee
		• Bovine serum albumen^39^
		• Wood particles
		• Evaporated milk
		• Corn syrup
		• Egg white
		• Vegetable oil
		• Human blood^40^
		• Chicken egg albumen^41^
		• Bacteriological agar
	Suture training	• Swine tissue
	NIR imaging	• Water
Simple block phantoms	PA imaging	• Nylon spheres^42^
		• Naphthol green dye
		• Silicone
		• Ethanol^43^
		• Agar^44^
		• Araldite resin
		• PVA
		• Epolight™ dye
	Optical scattering/absorption mimicking	• ${\mathrm{TiO}}_{2}$^45^
		• Intralipid^46^
		• Hydrogels^47^
		• Graphite
	Ultrasonic medical imaging	• Silicone
		• Graphite
		• Silica particles
		• Agarose
		• Agar
		• Talc^48^
		• ${\mathrm{Al}}_{2}O_{3}$
	MRI	• Carrageenan gel^49^^,^^50^
		• Agarose

### 2.1 Simple Tube/Channel Phantoms

**Fig. 2:**
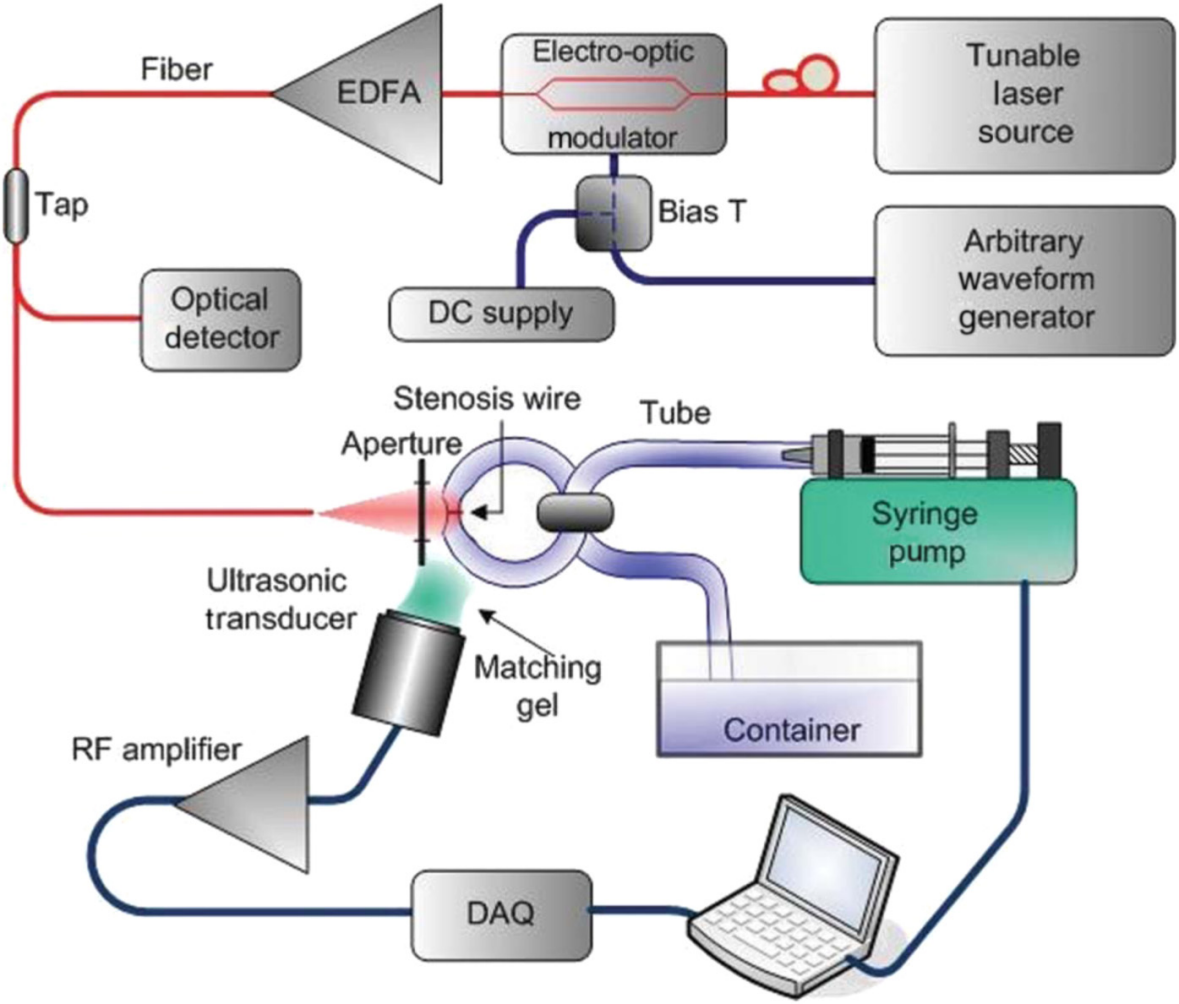
An image containing a simple tube phantom. A tube full of fluid was pulsed with a syringe pump and collected in a container. The flow of the fluid through the tube could be measured using PA.

Simple tube/channel phantoms are categorized as devices comprising a single tube or channel. Fluid or air can be pulsated through the tube using a pump, and it can be collected in a reservoir located at the tube’s end. Tubing materials are frequently employed due to their ability to simulate the structure of arteries or veins, and their behavior can be modeled using 3D software programs, such as Flow 3D^51^ or similar software. The tubing material can be tailored to achieve specific responses by choosing a high or low Young’s modulus. Simple tube phantoms have found application in various biomedical domains.^33^^,^^34^ Tubes and channel phantoms are frequently utilized in the field of acoustics.^52^^–^^54^ With the aid of B-mode ultrasound, a comprehensive cross-sectional measurement of a specific tube or channel can be obtained. These cross-sectional data enable the extraction of essential parameters, such as flow characteristics and microbubble formation. Figure 2 shows an instance where a tube phantom is employed for flow measurements.

In this study, a rubber tube was subjected to simulated stenosis by introducing multiple metal wires at various pressure settings. The stenosis wire-induced alterations in the tube diameter resulted in changes in the flow rate of the optically absorbent simulant fluid, which were measured using PA imaging. The flow was actively controlled to achieve flow rates of up to 55  mL/min with a syringe pump, serving as a reference for comparing and validating the PA Doppler flow rate measurements. The Doppler flow was measured through a tunable PA setup.^34^

### 2.2 Naturally Occurring Phantoms (Simple)

**Fig. 3:**
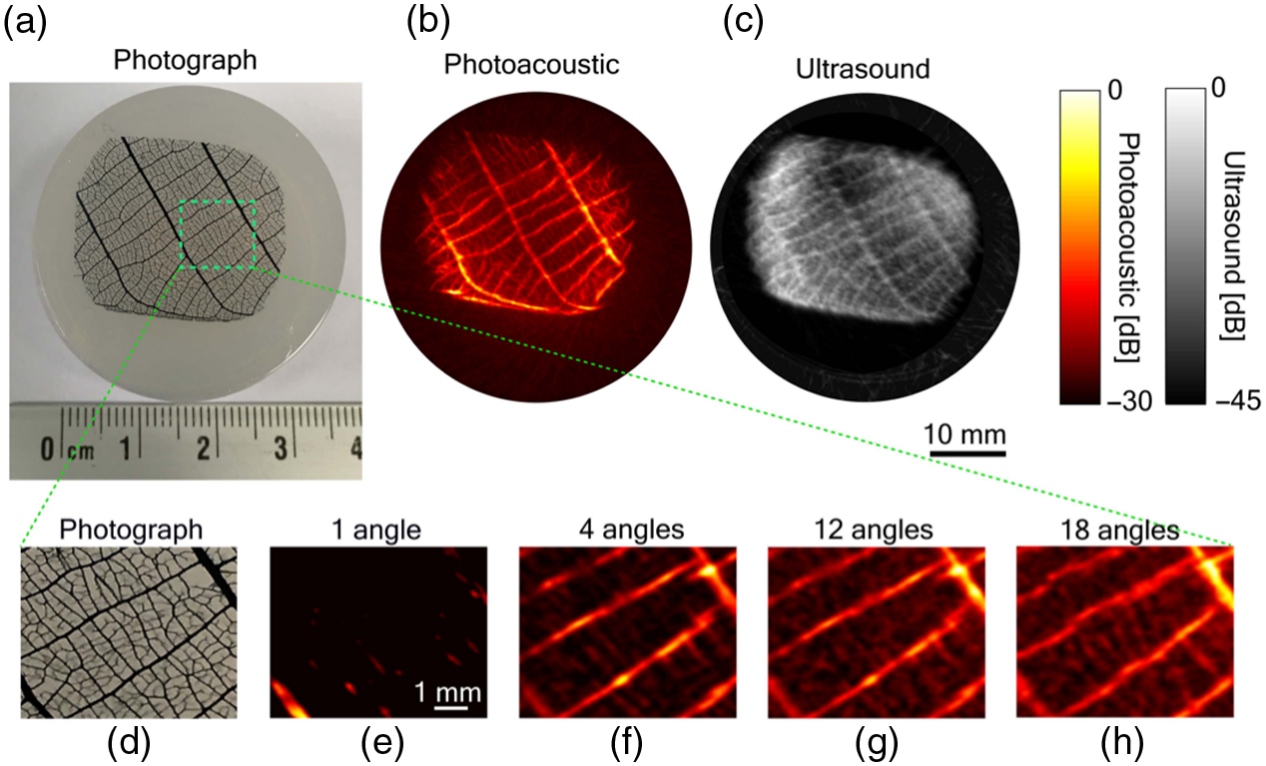
An example of a simple naturally occurring phantom. Francis et al. (Ref. 36) image the leaf phantom with three different methods: (a) optical photograph, (b) PA, and (c) ultrasound. (d)–(h) The resolution of PA was compared to the photograph when the number of angles was changed.

Numerous test phantoms can be derived from naturally occurring materials, whereby the structural components are obtained from natural resources or living organisms. In the context of the simple category, minimal or no manufacturing processes are required to prepare a naturally occurring phantom for utilization with a medical imaging sensor. However, certain structures may necessitate the addition of absorption media after harvesting to enhance result clarity. One common application of the naturally occurring sub-category is the utilization of leaves for quantifying the resolution of PA systems. Leaves possess veins of varying sizes and shapes that traverse the leaf structure, which can be measured and compared to the vasculature in the human body. By using different inks to pigment the structure, the PA effect can be generated specifically from the leaf veins.^36^^–^^38^ Figure 3 shows an example of a simple naturally occurring phantom. The leaf functions as a calibration tool to evaluate the image quality of the ground truth system, which is represented by an optical photograph, and other technologies investigated, namely ultrasound and PA imaging. Figures 3(a)–3(c) show the stock images obtained from all three imaging systems, captured at their respective highest resolution settings. Subsequently, a magnified section of the photograph is compared to a PA image of the leaf captured from the corresponding region, employing multiple angular views, specifically 1, 4, 12, and 18 angles in Figs. 3(d)–3(h). With an increase in the number of angles used in the PA system, smaller details that are discernible in the photograph become resolved. The leaf serves as an effective subject in this study due to its varied line thickness and intricate vein structure, contributing to favorable performance in terms of image resolution.^36^

In various applications, biological components derived from animals are employed.^55^^,^^56^ For instance, Ali et al. utilize chicken breast tissue implanted with pimento olives and knox gelatin embedded with blueberries to simulate tissue with lesions. These phantoms serve as training tools for ultrasound technicians and surgeons, allowing them to practice lesion localization through ultrasound imaging and needle biopsy techniques prior to performing these procedures on actual human patients. These materials are particularly advantageous in this application because they closely mimic the soft tissue characteristics of the human body surrounding a lesion. In addition, various animal tissues, such as swine, lamb, and cow tissues, find utility in the medical field for medical training purposes.^57^ Their similarities to human tissue afford surgeons a more realistic material for honing their skills in areas, such as suturing, medical imaging, and performing invasive surgeries.

Jiang et al. conducted a study where human blood was employed in the fabrication of human breast phantoms. These phantoms were infused with oxygenated blood and subjected to near-infrared (NIR) imaging, enabling the assessment of repeatability in measuring hemoglobin and oxygen levels.^40^ Numerous biological materials can be utilized as benchmarks in PA imaging studies.

### 2.3 Simple Block Phantoms

The simple block phantom is widely preferred in various applications owing to its straightforward manufacturing process and customizable nature. This type of phantom is characterized by a solid, single-layer, homogeneous block or disk, typically fabricated from a rubber base. To further tailor the properties of the material, scattering centers and dyes can be incorporated into the rubber mixture. Block phantoms are commonly designed to match specific parameters or anatomical locations within the human body. Block phantoms find widespread utilization in MRI for various medical and cancer imaging applications.^49^^,^^58^ In a study conducted by Hattori et al., the application of simple block phantoms is examined specifically for three Tesla MRI.^49^ The paper highlights the advantages of developing 19 distinct block phantoms designed to replicate the $T_{1}$ and $T_{2}$ relaxation times of multiple human tissues, thereby facilitating more accurate tissue characterization in MRI imaging. Hattori’s work outlines the commonly employed tissue materials in MRI phantoms, which typically consist of agarose, agar, PVA, gelatin, and polyacrylamide as base materials. These materials are combined with relaxivity-mimicking ions, such as ${\mathrm{CuSO}}_{4}$, ${\mathrm{NiCl}}_{2}$, ${\mathrm{MnCl}}_{2}$, and ${\mathrm{GdCl}}_{3}$. This comprehensive characterization forms the foundation for the development of multi-layered phantoms capable of representing various physiological tissues in MRI imaging.

PA phantoms can leverage a wide range of materials utilized in simple block MRI phantoms. Gelatin, a common base material employed in MRI phantoms for controlling relaxivity, can also be used as a tissue-mimicking material in PA phantoms.^59^ By adopting a similar manufacturing process, gelatin can serve as a suitable matrix for incorporating absorbing and scattering particles, thereby facilitating the development of PA phantoms with desired optical properties.^60^ The optical and acoustic properties of gelatin can be tailored to fulfill specific application requirements, and established methods are available for this purpose.^60^^,^^61^ Once the desired material properties of gelatin are determined, the addition of ${\mathrm{TiO}}_{2}$ and carbon black powder at varying concentrations allows for the attainment of suitable optical scattering and optical absorption characteristics. ^45^^,^^62^^,^^63^ The addition of ${\mathrm{Al}}_{2}O_{3}$ allows for the precise control of the acoustic absorption and scattering.^39^

**Fig. 4:**
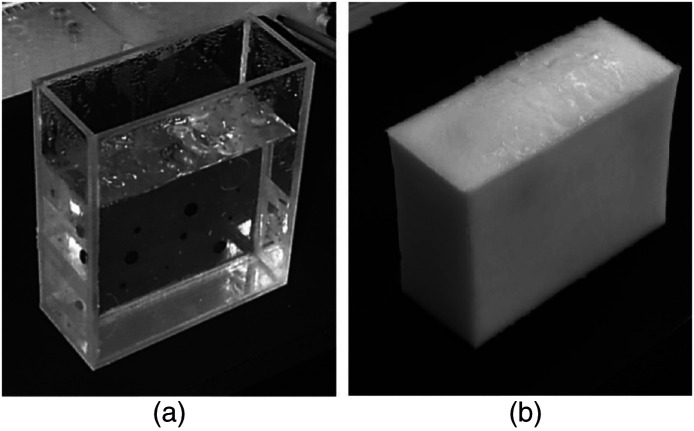
A simple block phantom is depicted. PVA was used as a base material. (a) The clear PVA with dark PVA gel spheres representing breast cancer. (b) The final block phantom after going through four freezing and thawing cycles.

Block phantoms are also widely employed in ultrasound for cancer imaging. In the study conducted by Manohar et al., a simple block phantom is constructed using polyvinyl alcohol (PVA) as the base material. Figure 4 shows the phantom developed by Manohar and the subsequent incorporation of embedded nylon spheres within a single layer of PVA to simulate the presence of tumors within the tissue. The dark color of the nylon spheres creates a noticeable contrast between the surrounding tissue and the spheres, enhancing their visibility during ultrasound imaging.^42^ In PA phantoms, various pigments can be incorporated into the material to achieve targeted optical absorption properties. Moffitt et al. utilized Epolight™ dye and titanium oxide to create block phantoms specifically designed for a particular wavelength of light used in PA imaging.^45^ By employing this approach, the resulting blocks effectively simulate human tissue at that specific wavelength, allowing for more accurate and realistic PA imaging experiments.

## 3 Intermediate Phantoms

Intermediate phantoms are characterized as having two to three major components and a moderate level of manufacturing complexity. They are further classified into three sub-categories: multiple layered block phantoms, embedded tube/channel phantoms, and naturally occurring phantoms (intermediate). This classification closely mirrors the differentiation observed within the simple category. Intermediate phantoms are relatively new in their application to PA imaging. For a comprehensive overview of all categories, associated materials, and their respective applications, refer to Table 3.

**Table 3:** Phantoms that exist in the intermediate category.

Category	Applications	Materials used
Embedded tube/channel phantoms	Ultrasonic imaging	• Ballistic gel^64^
		• PVC
	Balloon and stent surgeon practice	• Wax^65^
		• Silicone
		• ${\mathrm{Al}}_{2}O_{3}$
		• Silicone tube^66^^–^^68^
	Intravascular ultrasound and PA	• PVA^69^
	Piezoelectric blood pressure prediction	• Mineral oil^70^
	NIR imaging	• Araldite GY502^71^
		• Epoxy
		• Indocyanine green
	Photodynamic therapy	• Wax^65^
		• Silicone
		• ${\mathrm{Al}}_{2}O_{3}$
		• Polystyrene microspheres
		• Graphite
Naturally occurring phantoms (intermediate)	Ultrasonic and PA biometrics	• Human fingerprint^72^
		• Methemoglobin
	NIR imaging	• Gelatin
		• Hemoglobin
		• Intralipid^73^
	PA imaging	• Chicken breast^35^
		• Gold nanoparticles
	Ultrasonic imaging	• Chicken breast
		• Agarose
		• Condensed milk
	MRI and computational tomography	• Safflower oil
		• Polyurethane mesh^74^
		• Agarose
		• Condensed milk
		• N-propyl alcohol
		• Sodium chloride
	Optical signatures associated with the dysplasia to carcinoma	• Collagen^75^
	High resolution molecular reflectance imaging	• Epithelial cervical cells^76^
		• Tri-buffered saline
	Optical time of flight imaging of phantom breast tissue	• Dairy free creamer^77^
Multiple layered block phantom	Optical absorption and scatter mimicking	• Nylon spheres
		• Polystyrene microspheres
		• Silica spheres
		• Oil based ink
		•${\mathrm{TiO}}_{2}$^78^
		• Quartz glass spheres
	Optical imaging	• PVA^79^
		• Latex
		• Epoxy
		• Cobalt
		• Methylethylketone^80^
	PA imaging	• Native gel wax^62^
	Thermal therapy and MRI	• Polyacrylamide gels
		• Acrylamide^81^
	Ultrasound attenuation/scattering mimicking	• Glass beads
	NIR imaging	• Polyester resin^82^^,^^83^
		• Peroxide

### 3.1 Embedded Tube/Channel Phantom

**Fig. 5:**
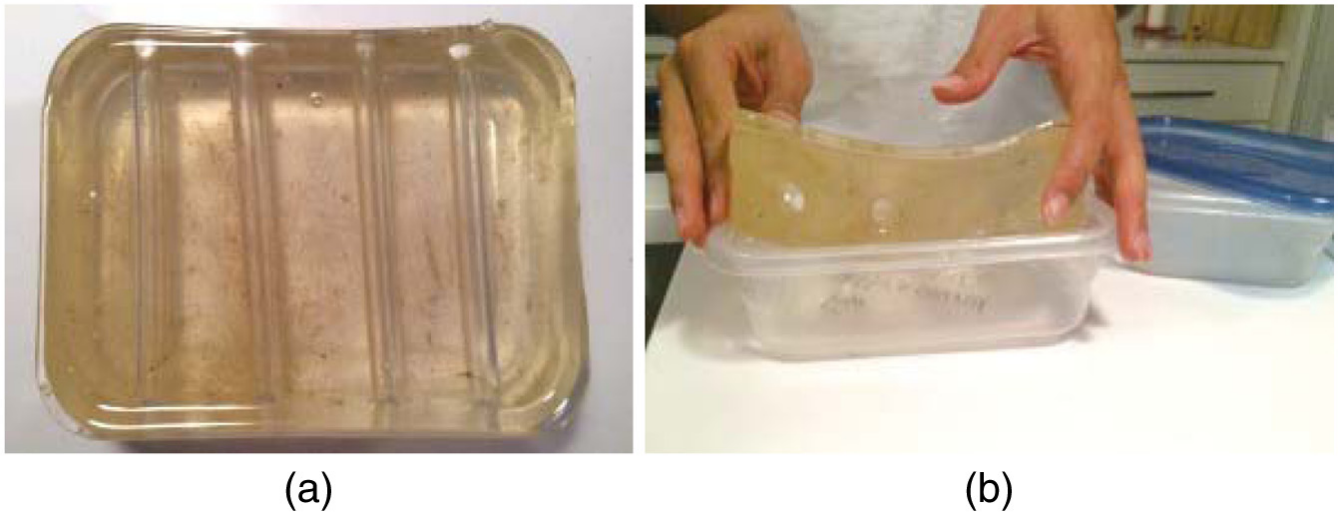
An example of an embedded tube/channel phantom. PVC pipe was used to create four channels within a ballistic gel. The phantom was then sealed and used for ultrasonic measurement. (a) A top view of the phantom and (b) the channels from the side.

Embedded tube/channel phantoms are categorized as phantoms consisting of a base material, typically rubber, that incorporates a tube or channel. These phantoms are commonly utilized to replicate the structure of veins or arteries within the body. They find utility in imaging modalities such as ultrasound and optics, which focus on specific vascular regions of interest. Figure 5 provides an example of such a phantom. This phantom comprises four cylindrical channels embedded within a block of ballistic gel. Each channel is filled with a fluid to simulate realistic vessel imaging under ultrasound. The materials used were carefully chosen to ensure simplicity, cost-effectiveness, and facilitate rapid manufacturing. This phantom facilitates the training of ultrasound technicians and doctors in patient injections by enabling hands-on, practice-based learning.^64^

In the field of optical imaging, phantoms have been developed to replicate the internal structure of the human bronchial vasculature.^65^ Bays et al. discuss the utilization of wax embedded in a silicone base to fabricate channels that mimic the bronchial vasculature. The desired vasculature pattern is initially molded using wax and then incorporated into a silicone material for stability and durability. The silicone material used in the phantom is carefully adjusted to replicate the optical scattering and absorption properties of human tissue, often achieved through the incorporation of materials, such as ${\mathrm{Al}}_{2}O_{3}$. Once the silicone is cured, the wax is dissolved, leaving behind an empty cavity that can be utilized for training purposes, specifically for stent and balloon placement. Given the phantom’s equivalent optical properties to the human body, surgeons can effectively image the phantom and practice stent or balloon placement under near real-life conditions.

In the study by Peng et al., a piezoelectric ultrasound system is employed in conjunction with a tube phantom connected to a pulsing syringe pump.^70^ By tracking the movement of the anterior and posterior walls of the pulsating tube phantom using ultrasound, the authors predict blood pressure. Similar methodologies can be directly applied to the field of PA imaging. PA imaging can leverage these phantoms to monitor the vasculature of cancerous tumors and track parameters, such as blood glucose and oxygen levels.^84^^–^^86^ The studies conducted *in vivo* could greatly benefit from the availability of an accurate PA channel phantom equipped with a blood simulant. By combining various properties of optics and ultrasound, it is possible to develop a tube phantom that closely mimics the PA properties of human tissue. Such a phantom would enable the quantification of PA imaging capabilities in terms of measuring vasculature at different depths, sizes, and locations within the body. This would enhance the understanding and assessment of PA imaging techniques for clinical applications.

### 3.2 Naturally Occurring Phantoms (Intermediate)

**Fig. 6:**
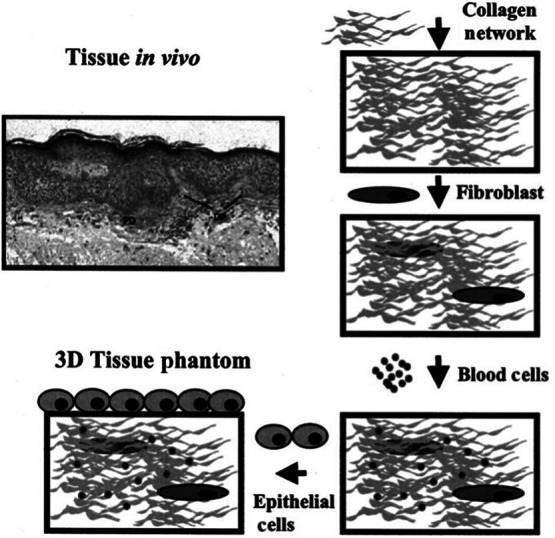
An example of an intermediate naturally occurring test phantom. A model tissue was used for a reference and is labeled “tissue *in vivo*.” A manufacturing process is then described by first creating a base of collagen. Fibroblasts were then added followed by blood cells. Finally, a layer of epithelial cells was dispersed on top to create the simulant tissue.

Intermediate naturally occurring phantoms are characterized by their moderate manufacturing effort and are composed of an underlying structure derived from living tissue or natural materials. Figure 6 presents an example of such a phantom. In this case, an epithelial phantom was developed to investigate the optical properties of human epithelial layers and the impact of developing carcinoma on these properties. The manufacturing process involves multiple steps, including the layering of collagen, fibroblasts, blood cells, and epithelial cells, to recreate the desired structure and composition. The optical imaging techniques employed in this study encompassed fluorescence microscopy, transmittance microscopy, and fluorescence excitation-emission matrixes. These methods were applied to both the tissue phantom and actual human cervical epithelial biopsies. The study aimed to investigate the variations in optical properties across different testing conditions, time intervals, and environmental factors that are typically encountered during optical biopsy scanning of the human epithelial. The findings shed light on the changes in optical properties and their implications for accurate diagnosis and monitoring of the human epithelial tissue.^75^ In a review article by Pogue et al., various natural materials are discussed for their application in fabricating optically equivalent tissue phantoms.^78^ Materials, such as milk, lipid, and intralipid, are utilized to match optical absorptions and introduce scattering characteristics into the phantoms.

The application of intermediate naturally occurring phantoms in the PA field is in its early stages.^35^^,^^69^^,^^72^ Further advancements are required for the development of PA test phantoms that incorporate natural properties. Phantoms, such as the one described by Sokolov et al., could be instrumental in testing the safety limits of PA imaging.^75^ As the field of PA imaging advances, there is a growing interest in investigating the safety of PA imaging on skin and organs.^87^^–^^89^ The utilization of naturally occurring phantoms that incorporate human cells could significantly reduce the necessity for human trials and expedite the preliminary testing of parameters such as wavelength and laser power concerning human skin and organ safety.

### 3.3 Multiple-Layered Block Phantom

**Fig. 7:**
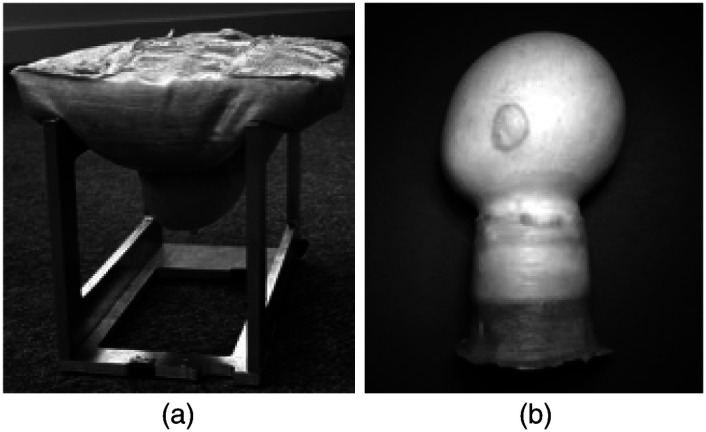
An example of two ML block phantoms. (a) A human breast phantom. The shape of the human breast was created through a flexible latex shell. The center of the breast was filled with a PVA liquid. (b) A human baby head phantom. This head was created with a latex shell and PVA interior. Scattering material could be added to the inside PVA material to represent cancer sites.

Multiple-layered (ML) block phantoms operate on a similar principle as simple block phantoms, but they introduce multiple layers of rubber or other materials to create a phantom with varying properties at different depths. The manufacturing process for ML phantoms involves intermediate-level procedures, such as curing and structural arrangement. ML phantoms are frequently utilized in the imaging field due to their applicability to a wide range of applications and experiments, as well as their ability to be manufactured in laboratory settings. ML phantoms are frequently employed to replicate specific regions of the body for cancer imaging purposes.^71^^,^^74^^,^^77^^,^^79^ In a study by Gibson et al., a series of ML phantoms were developed to simulate a baby’s head and a woman’s breast.^79^ The fabrication involved encapsulating a mixture of fluidic PVA, microspheres, and dye within a latex shell, which was then molded into the shape of a human breast. The resulting phantom enabled imaging comparisons with real human breast tissue using both x-ray and optical imaging modalities. Figure 7 shows an ML block phantom, as classified in this context. Pogue et al. developed breast phantoms with realistic tumors for NIR imaging applications.^71^ The study employed Araldite rubber as the base material, with liquid epoxy resin used to simulate tumors within the phantom. NIR images of the phantom were then compared to those of actual human breast tumors. ML phantoms are commonly employed to replicate specific tissue layers within the body, aiming to mimic various components, such as skin, human tissue, and organs.^49^^,^^60^^,^^80^^–^^82^^,^^90^^,^^91^

## 4 Advanced Phantoms

Advanced phantoms are categorized into three sub-categories: advanced tube/channel (ATC) phantoms, advanced layered phantoms, and test target phantoms. These phantoms require extensive manufacturing efforts and time as they incorporate four or more different components. They serve as versatile testing tools for a wide range of applications. Currently, there are limited advanced phantoms available specifically designed for PA imaging, indicating a significant opportunity for further development in this area. For a comprehensive overview of advanced phantom categories, associated materials, and their respective applications, please refer to Table 4.

**Table 4:** Phantoms that exist in the advanced category.

Category	Applications	Materials used
ATC phantoms	MRI fluid dynamics	• Polylactic acid (PLA) plastic^92^
		• CAGN gel^93^ (gel mixed from carrageenan, agarose, ${\mathrm{GdCl}}_{3}$, ${\mathrm{NaN}}_{3}$, NaCl)
	PA imaging	• PLA plastic^22^^,^^94^
	Pulse wave propagation tracking	• Silicone tubes^4^
		• Polyacrylamide^95^
	Respiratory and cardiac motion detection	• Silicone rubber^96^
	Surgical training	• TangoPlus^1^^,^^2^
Advanced layered phantom	Deep tissue injury simulation	• Human skeletal model^97^
	Facial spoofing	• PLA plastic^3^
		• Dragon skin
		• Ecoflex 0010
		• Ecoflex 0030
		• Injection molded plastic^98^
	Photodynamic and fluorescent spectroscopy	• Sodium azide
		• Bovine blood syrum^99^
		• Drawing ink
	PA imaging	• PVA^30^
	MRI imaging	• RGD-525, Stratasys ©^5^
	Optical sectioning	• Cervical cells
		• Collagen^100^
	Prostate radiation therapy	• Perspex^®^
		• Gold^101^
	PA and ultrasonic breast simulation	• Computer simulation^102^
Test target phantoms	PA resolution quantification	• PDMS^103^
		• Silicone oil
		• DI water
		• ${\mathrm{TiO}}_{2}$
		• Polyacrylamide
		• Carbomer^104^
		• Intralipid^105^^,^^106^
	MRI resolution	• Polyvinylpyrrolidone solutions^107^
		• Gadolinium
		• Agarose
		• Polystyrene spheres^91^
		• PMMA
		• Polyoxymethylene^108^
	Optical imaging	• Polyurethane^109^
	PET computational tomography resolution	• Cell foam^91^
		• Alginate
		• Radioisotope

### 4.1 Advanced Tube/Channel Phantoms

An ATC phantom is characterized by the presence of multiple intricate channels or tubes embedded within a base material at different depths. These channels or tubes can effectively simulate various aspects of human vasculature at multiple depths, cavities within the heart, and even respiratory behavior. ATC phantoms offer broad applications and can be utilized in numerous contexts requiring the representation of complex anatomical structures and physiological behaviors. Hacham et al. employed silicone tubes submerged in a water bath to simulate stenosis and aneurysm conditions experienced by patients.^4^ The experiment involved the use of a reciprocating pump to drive fluid through the tube, with continuous monitoring of flow rate and pressure using dedicated probes. The aim of this experiment was to replicate the behavior of blood flow and pressure when flow is induced in an artery affected by stenosis or aneurysm. The design of the silicone tubes was specifically tailored to mimic the deformations associated with these pathological phenomena.

**Fig. 8:**
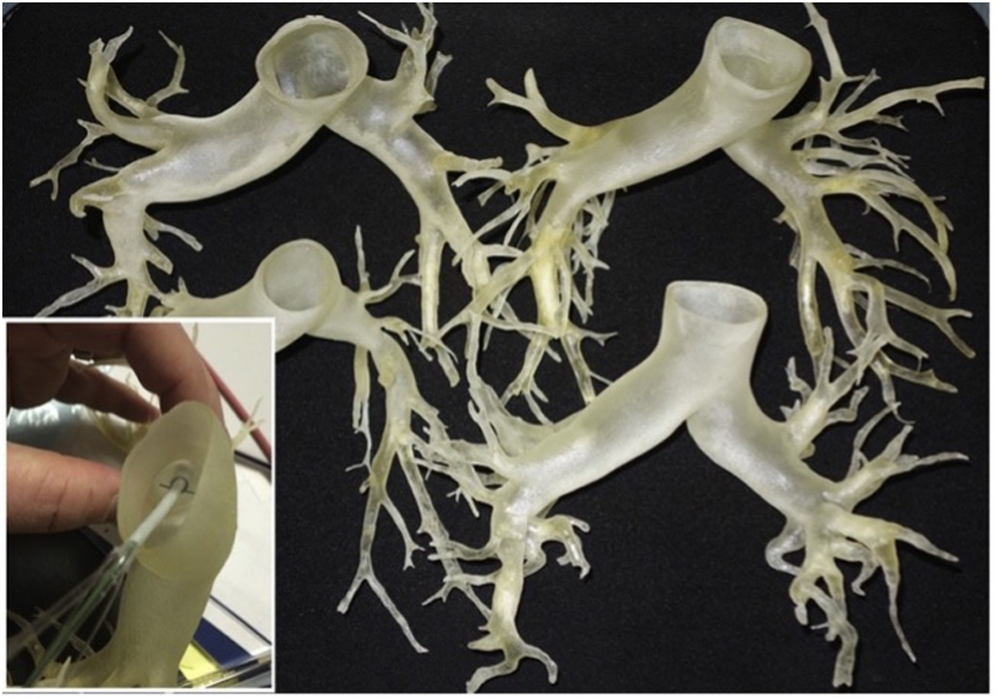
An example of an ATC phantom. This phantom mimics the human pulmonary artery. This phantom was 3D printed using a PolyJet^©^ printer. The bottom left hand of the image shows an example of a catheter being placed into the phantom.

3D printing has brought about a transformative change in the fabrication of ATC phantoms.^1^^–^^3^^,^^22^ Kurenov et al. discuss the utilization of 3D printing technology to create neurovascular models using a flexible material called TangoPlus^©^.^1^ Through the use of computerized tomography (CT) scans, accurate replicas of patient-specific anatomical structures in terms of size and morphology are generated. These phantoms serve as tools for surgeons to practice thoracic surgery and perform procedures, such as stent retrieval and removal. By simulating patient-specific scenarios, these phantoms enable surgeons to gain essential preoperative practice, contributing to improved surgical outcomes. Figure 8 showcases the phantom utilized by Kurenov et al., demonstrating its practical application in neurovascular modeling. It is worth noting that PA phantoms currently available in the ATC category do not exhibit the advanced structural complexity depicted in Fig. 8.^22^^,^^94^ By adopting the manufacturing process outlined by Kurenov et al., it is possible to fabricate an advanced 3D printed PA phantom. However, before utilizing TangoPlus as the phantom material, it is crucial to analyze its acoustic and optical properties to assess its performance in PA imaging. Several research papers have investigated the speed of sound, acoustic impedance, and acoustic attenuation of TangoPlus.^56^^,^^110^ However, a major challenge lies in determining the optical properties of TangoPlus, as there is currently a scarcity of literature in this specific area. To overcome this limitation, users would need to quantify the absorption and scattering coefficients of the stock material to better understand its optical behavior in the context of PA imaging. Unlike other PA phantom materials, the adjustability of optical properties in TangoPlus or similar 3D printing media is limited, as it is challenging to incorporate common scattering and absorption materials during the printing process. However, it is possible to create a surrounding tissue for the vascular network using a combination of water and lipid, which can be tuned to achieve desired scattering and absorption coefficients.^6^^,^^105^ In addition, by utilizing a peristaltic pump as employed by Kurenov et al., a blood simulant with acoustic and optical properties can be produced. This simulant can be based on a mixture of water, glycerol, and dextran and further enhanced with the inclusion of 10  μm polyamide microspheres. This combination of materials enables the development of a realistic blood-mimicking medium with appropriate acoustic and optical characteristics for use in PA imaging experiments.^1^^,^^63^

### 4.2 Advanced Layered Phantom

**Fig. 9:**
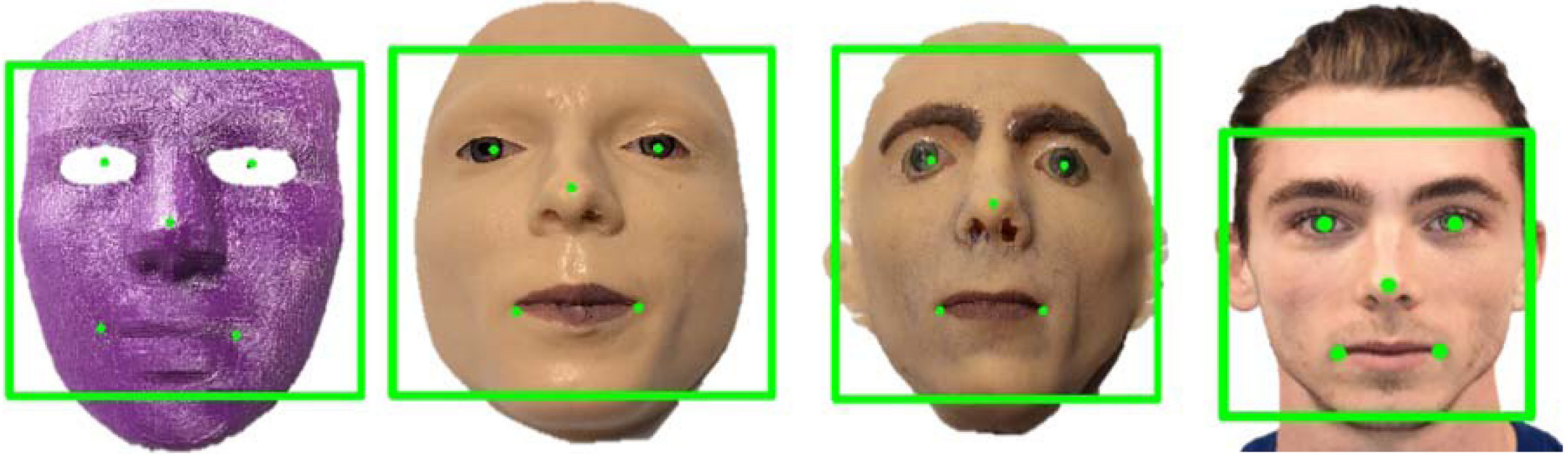
An example of an advanced layered phantom. Three different phantoms are depicted. On the far left, a 3D-printed phantom face is created from a 2D image reconstruction. Both phantoms in the middle are created using EcoFlex 0030 and Dragon skin rubber. They have layers for different features of the face. This phantom was then used to spoof the iPhone Face ID technology.

Advanced layered phantoms are characterized by the presence of four or more components or layers, necessitating extensive manufacturing efforts and time. These phantoms are designed to replicate specific regions of the body, encompassing an entire structural composition. Figure 9 showcases an example of a layered phantom representing a human face. Face ID is a prominent feature introduced by Apple in the 10th generation iPhone. Christie et al. discuss the creation of phantoms for spoofing face identification technology using two methods: live casting of a human face and 3D printing a face based on a two-dimensional (2D) image. The face-layered phantoms serve the purpose of simulating human faces and are employed in assessing the effectiveness of face recognition systems. Comparisons were made between the thermal properties of the plastic and rubber used in the phantoms and those of actual human faces. Through the use of these phantoms, the study proposes improvements to the optical scanner in order to enhance iPhone security and prevent unauthorized access.^3^ By employing a similar manufacturing process, substitutes to EcoFlex 0030, such as polydimethylsiloxane (PDMS), can be utilized as a rubber material for creating phantoms with tuned optical and acoustic properties resembling those of the human face. This would enable the evaluation of PA imaging on regions of the face that are typically not measured due to concerns about eye safety associated with high-power laser exposure.

MRI has widely employed advanced layered phantoms, and Sun et al. provide insights into the creation of a 3D pelvic phantom designed for MRI imaging.^5^^,^^101^^,^^111^ This phantom is constructed using a Perspex® plastic shell and is filled with gold microbeads or sheets to mimic the characteristics of the human prostate. By manipulating the filling with either fluid or leaving it exposed to air, the phantom can simulate various pelvic organs. This innovative approach holds the potential to expedite the development of MRI techniques for detecting prostate cancer, eliminating the need for human subjects during early testing stages. The realistic representation of pelvic organs provided by this phantom contributes to advancing MRI technology in the field of prostate cancer diagnosis and aids in refining imaging protocols and techniques. This phantom exhibits promising potential for adaptation within the PA imaging domain. The optical and acoustic properties, specific to polymethylmethacrylate (PMMA) plastic, have been comprehensively documented by multiple research groups.^112^^,^^113^ Substitutions may be implemented in the fluid utilized to fill the primary chamber, as well as the smaller chambers representing anatomical structures, such as the human prostate, rectum, bladder, and femoral heads. D’Souza et al. provide a comprehensive overview of the evaluation of diverse tissue-mimicking materials for a prostate phantom that incorporates ultrasonic and relaxivity properties consistent with different regions of the human prostate.^74^ To achieve the desired acoustic attenuation, speed of sound, and backscatter characteristics in the target region, the ratios of condensed, agarose, and glass beads with varying sizes were carefully adjusted. In addition, PA phantoms were developed using an agarose base, with the inclusion of gel wax and black oil color to precisely control the optical absorption and scattering coefficients.^114^ To create a more accurate prostate phantom, the user should begin by obtaining the optical properties specific to the prostate tissue. This information can be gathered from relevant literature or experimental measurements. Subsequently, the quantity of wax and black oil should be adjusted accordingly to achieve optical properties that closely resemble those of the prostate. When constructing the phantom, it is crucial to ensure that all chambers are filled with tissue-mimicking materials as acoustic signals are unable to transmit through air.

Phantoms have been developed to replicate the characteristics of deep-tissue injuries as well. Sparks et al. utilized various silicone rubbers and bone simulants to construct a human thorax phantom.^97^ The shear and hyperelastic moduli of the thorax were quantified through an indentation test using a load cell. In addition, the strain across the silicone rubber was measured, accurately reflecting the behavior of the human thorax under similar conditions.^97^ There is a scarcity of phantoms specifically designed for PA imaging in the advanced layered category. However, methodologies such as Sparks et al. can be employed within the PA domain to fabricate phantoms that possess comparable optical, acoustic, and mechanical properties to those found in the human body. By incorporating such techniques, it becomes feasible to create phantoms that accurately emulate the complex nature of layered tissues for PA imaging applications.

### 4.3 Test Target Phantoms

Test target phantoms represent a distinct category of phantoms within the advanced classification, primarily due to their intricate design and purpose. These phantoms are specifically designed to assess the resolution capabilities of different imaging modalities. While test targets are commonly used in the optical domain to verify the resolution of cameras and microscopes, they have also found applications in other technologies for resolution quantification. Typically, test targets consist of a defined set of line pairs per millimeter (lp/mm) that serve as reference patterns to quantify the resolution of an imaging system.^115^ They have been extensively employed in the optical field, but their utility extends beyond that. For instance, Büchele et al. utilized a test target phantom to evaluate the resolution of an x-ray imaging technique. This demonstrates the adaptability of such structures across various imaging modalities for the purpose of resolution measurement and assessment.^116^

MRI imaging heavily relies on the relaxation times ($T_{1}$ and $T_{2}$) and magnetic field ($B_{0}$) properties of materials. Keenan et al. documented the development of a brain imaging phantom specifically designed to simulate these properties for MRI testing.^107^ The phantom consists of 32 individual spheres, each capable of being filled with a material that mimics specific properties. In their experiment, Keenan et al. manipulated the concentrations of ${\mathrm{NiCl}}_{2}$ within the spheres to generate multiple relaxivity (R) measurements. By varying the relaxivity values, different resolutions could be defined and characterized. This approach facilitates the assessment and calibration of MRI techniques, ultimately contributing to the advancement of image resolution in brain imaging studies. Although ${\mathrm{NiCl}}_{2}$ is not widely used in the PA imaging space, a similar phantom structure can be used when creating a phantom for PA. The spheres can be filled with acoustic and optical materials, such as ${\mathrm{Al}}_{2}O_{3}$, ${\mathrm{TiO}}_{2}$, and carbon black powder in different concentrations. This allows for the tuning of sensor optical/acoustic abosprtion, optical/acoustic scattering, speed of sound, and acoustic attenuation.

**Fig. 10:**
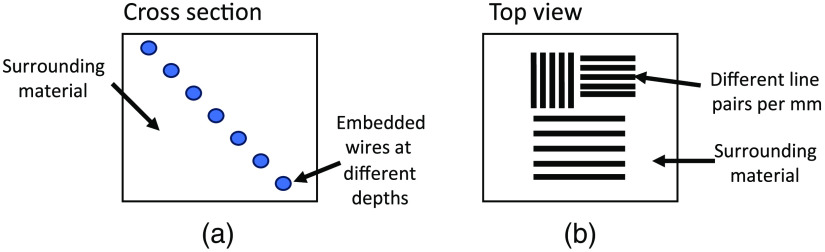
Two phantom cartoons that can be implemented for PA imaging. (a) The cross section of a phantom containing a series of wires embedded in a surrounding tissue. The wires are a set diameter and allow for a quantification of resolution as a function of depth. (b) A lp/mm phantom embedded in a surrounding tissue. This phantom allows for the quantification of elevation and lateral resolutions.

In the field of PA imaging, Palma-Chavez et al. employed similar methodologies.^104^ Their manuscript details the fabrication of a phantom that incorporates targets of varying sizes and optical absorption properties at different depths within a tissue-mimicking material. The imaging targets were generated using 3D printing technology, with diameters ranging from 1 to 4 mm. These targets were then embedded at multiple depths within the phantom. The tissue-mimicking material was developed by combining cultured fat/parenchyma cells, titanium dioxide, silicone oil, DI water, ethylene glycol, and tween 20 surfactant. Like Keenan’s approach in MRI phantom testing, this PA phantom enables the evaluation of various PA parameters, including depth penetration, optical absorption, resolution, and optical scattering. Previous studies have described the use of different lp/mm structures to evaluate the axial, lateral, and longitudinal resolutions of PA sensors.^103^^,^^105^^,^^106^ Vogt et al. utilized a PA imaging setup to quantify the image performance of their PA system. The PA test target phantom was created by implanting metal wires at various depths within a base material of polyvinyl chloride plastisol (PVCP). To achieve desired optical and acoustic properties, the PVCP was fine-tuned using ${\mathrm{TiO}}_{2}$, glass microspheres, and black plastic colorant. By incorporating these materials, the phantom’s optical and acoustic characteristics could be precisely controlled.^106^ A similar phantom cartoon is shown in Fig. 10(a), which can be used to detect the depth resolution of an imaging system. This phantom has multiple wires embedded in an arbitrary surrounding tissue. Figure 10(b) shows a phantom comprised of lp/mm in an arbitrary material that can quantify a PA lateral and axial resolution. With further advancements in the field, target phantoms can become more tailored to PA characteristics. They can not only quantify resolutions, as mentioned above, but also enable the quantification of specific parameters such as absorption, scattering, attenuation, and speed of sound in different materials.

## 5 Discussion

The field of PA imaging is currently in its early stages of development, but it is expected to experience continuous growth in the foreseeable future. Several challenges in research and development have prevented the technology from becoming mainstream. To overcome these challenges, it can be beneficial to draw insights from the development of test phantoms in other well-established imaging modalities, such as MRI, optics, ultrasound, positron emission technology (PET), and CT. By studying the patterns observed in the development of test phantoms in these fields, valuable knowledge can be applied to the PA imaging domain with minimal modifications, thereby accelerating the commercialization process. As the development of phantoms progresses from simple to intermediate and then to advanced categories, the number of phantoms being specifically designed for PA imaging decreases. It would be advantageous to establish standard phantoms in each of the three advanced phantom categories: test target phantom, ATC phantom, and advanced layered phantom. These standardized phantoms, tailored to specific PA applications, can significantly streamline the commercialization efforts and facilitate further advancements in the field.

### 5.1 Test Target PA Phantom

A test target phantom plays a crucial role in quantifying system resolution and can greatly contribute to the advancement of the PA imaging field. One area where a test target phantom would prove beneficial for the development of PA imaging technology is in the domain of biometric sensing. Biometric sensors rely on accurate and reliable measurements, often requiring government certifications to ensure their effectiveness. NISTIR 7123 outlines the technology evaluation of fingerprint matching, identification, and verification. Usually a test target consisting of lp/mm like Fig. 10(b) is used to conduct this evaluation. Similar methods of resolution quantification can be employed when developing new biometric sensors.^117^ Zheng et al. already begun applying PA imaging to the biometric field, specifically in the context of liveness detection.^72^ Using a human hair with known dimensions, the lateral and elevation resolutions were determined. However, relying solely on human hair for resolution assessment poses challenges due to the inconsistency in hair diameter, making repeatability by others difficult. To address this limitation and achieve a more concrete definition of resolution in both the lateral and elevated planes, the implementation of a test target with precisely known dimensions would be more suitable.

Efforts are underway to develop test target phantoms, as evidenced by the works of various research groups.^36^^,^^103^^–^^106^ These phantoms serve the purpose of precisely defining the resolution of a system, although further advancements are still required. Schneider et al. introduced a microfluidic test target specifically designed for quantifying resolution in PA imaging.^103^ This phantom incorporates a microfluidic network with lp/mm, combining concepts from the PA field with optical test targets. By utilizing this phantom, the resolution capabilities of a chosen PA system can be determined. However, the phantom described by Schneider et al. lacked certain essential elements crucial for PA imaging, including a scatter/absorption layer preceding the target and variations in target depth. Incorporating a scatter/absorption layer before the target helps replicate the optical properties of biological tissues while introducing variations in target depth enables the assessment of imaging capabilities at different tissue depths.

### 5.2 Advanced Layered PA Phantom

The advanced layered PA phantom offers significant benefits in the development of PA imaging. Unlike test target and tube/channel phantoms, layered phantoms provide a higher level of detail and complexity. This is particularly valuable when designing PA imaging systems for training purposes, cancer imaging, and simulation, as these applications require realistic phantom features, such as tumors or layered skin structures. Currently, there are ongoing efforts to develop simple layered phantoms specifically tailored for PA cancer imaging.^42^^,^^47^ These phantoms are fabricated using straightforward manufacturing techniques and consist of single layers. Microbeads or microgels are incorporated into the base material to replicate cancerous tumors. MRI phantoms have been successfully developed for cancer imaging across the simple, intermediate, and advanced categories. For instance, Sun et al. constructed a prostate phantom for MRI imaging that incorporated multiple reservoirs to simulate different layers of the body and the human prostate.^101^ A similar shell-based approach could be adopted for PA imaging, utilizing a filler rubber material with appropriate optical absorption and scattering centers to closely mimic the optical properties of the human body.

Phantoms play a vital role in achieving accurate simulations and providing a consistent reference for testing parameters. Lou et al. employed simulations to generate images of a human breast phantom using PA imaging.^102^ A realistic breast phantom was simulated, incorporating layers, shape, and vasculature based on an MRI image. A simulated PA testing environment was then established to image the phantom file, and the results were compared to the original MRI image. However, this study did not include a comparison of the simulated results with a real test phantom, which could further validate the accuracy of the simulation. Clinical testing on human subjects can pose challenges, as data replication by other groups and concerns regarding reliability and repeatability can arise. To address these issues, a phantom breast can be developed and imaged using PA imaging techniques. The phantom can then be modeled in the same software as used by Lou et al., enabling a comparison between real PA images and simulated models. Variations can be analyzed and addressed to enhance the accuracy of the simulations. Methods employed by Hebden et al. can be utilized to create a breast phantom that closely mimics the structural characteristics of the human breast.^79^

### 5.3 Advanced Tube/Channel PA Phantoms

ATC phantoms play a pivotal role in advancing research and development in the field of PA imaging technology, particularly in areas, such as liveness detection, vascular imaging, and surgeon training. These phantoms enable the accurate replication of human vasculature by embedding it within a surrounding material, providing a realistic representation of anatomical structures. Liveness detection, a growing field in biometrics, focuses on distinguishing biometric measurements obtained from living humans from spoof attacks, which involve mimicking specific biometric features, such as the face or fingerprint.^118^ The vascular system in the human body provides valuable physiological features, such as heart rate, which can be captured and analyzed using PA imaging techniques to identify living individuals. The development of test phantoms that accurately mimic the human vasculature holds great potential for advancing liveness detection methodologies. By leveraging 3D printing technologies, it becomes possible to create a comprehensive model of the arterial vasculature based on CT or MRI images.^1^^,^^92^ This 3D printed vascular structure can be embedded within a tissue-mimicking material with optical and acoustic properties equivalent to human tissue. Such a phantom would not only contribute to the development of liveness detection techniques but could also serve as a valuable tool for surgeon training and improving the accuracy of procedures such as stent placement.

Vascular imaging in the field of PA imaging can greatly benefit from the availability of an ATC phantom. Specifically, in the context of cancer angiogenesis detection, PA imaging can be utilized to identify the formation of blood vessels that connect tumors to surrounding organs, a process known as angiogenesis.^119^ To effectively detect angiogenesis, the PA imaging device must be capable of identifying the newly developed capillary network within the tumor and distinguishing these vessels from other blood vessels in the body. A PA phantom similar to the one described by Christie et al. could be developed to mimic the blood vessels with diameters similar to those found in tumor capillaries.^22^^,^^94^ Additional arteries and veins could be incorporated within the phantom to assess the capability of PA imaging in distinguishing between veins, arteries, and cancerous capillaries based on their depth, diameter, and shape. Similar approaches can be employed to create vascular test phantoms for imaging various regions of the body, including the brain, arm, wrist, and neck.

## 6 Conclusion

Test phantoms play a crucial role in the development and commercialization of imaging technologies, enabling researchers to evaluate and optimize the performance of these technologies. Various imaging modalities, including MRI, CT, PET, ultrasound, and optics, have successfully utilized test phantoms of different complexities to advance their respective fields. By studying test phantoms used in other modalities, valuable insights regarding materials, methods, and structures can be gained, accelerating the development of test phantoms for new modalities such as PA imaging. An illustrative example of the impact of test phantoms on technology development is seen in the field of MRI, which has evolved from sub-1 Tesla capabilities to greater than 7 Tesla.^120^

The development of advanced test phantoms in various subcategories (tube/channel, layered, and test target) for PA imaging holds great potential for accelerating the progress of this imaging technology across multiple applications. By incorporating advancements and complexities observed in phantoms used in other commercial imaging modalities, PA can benefit from existing knowledge and methodologies, thereby expediting its development process. This cross-pollination of ideas and techniques can help address the current limitations and challenges faced in PA test phantoms.

The implementation of advanced test phantoms in PA can have a significant impact in several fields. In liveness detection and biometrics, the use of phantoms that accurately mimic the vasculature and other features of the human body can enhance the development of techniques for distinguishing between living subjects and spoof attacks. In vascular imaging, the availability of test phantoms that replicate the complex vascular networks of tumors and surrounding tissues can enable the evaluation and optimization of PA imaging for cancer angiogenesis detection. Similarly, in surgeon training, the use of realistic phantoms can provide a platform for practicing procedures such as stent placement in a safe and controlled environment. Furthermore, advanced test phantoms can aid in the development of PA imaging for cancer imaging, enabling the assessment of imaging parameters and optimizing the detection and characterization of cancerous tissues. The development of high-resolution phantoms can enhance the evaluation and improvement of PA system performance in terms of resolution and image quality. The creation of test target phantoms allows for the precise evaluation and calibration of PA systems, facilitating standardization and benchmarking in PA imaging research and clinical applications. All in all, by investing in the development of advanced test phantoms across different subcategories, PA imaging can benefit from accelerated progress in a wide range of applications, leading to advancements in general medical imaging and improving patient care.
